# Genetic Dissection of Milled Rice Grain Shape by Using a Recombinant Inbred Line Population and Validation of *qMLWR11.1* and *qMLWR11.2*

**DOI:** 10.3390/plants13223178

**Published:** 2024-11-13

**Authors:** Liting Zhang, Zhanhua Lu, Zhaoyang Pan, Tengkui Chen, Shiguang Wang, Wei Liu, Xiaofei Wang, Haoxiang Wu, Hao Chen, Yunyi Zhan, Xiuying He

**Affiliations:** 1 Rice Research Institute, Guangdong Academy of Agricultural Sciences, Guangzhou 510640, China; zhliting1018@163.com (L.Z.); luzhanhua@gdaas.cn (Z.L.); 15238535392@163.com (Z.P.); chentengkui@gdaas.cn (T.C.); wangshiguang@gdaas.cn (S.W.); liuwei@gdaas.cn (W.L.); wangxiaofei@gdaas.cn (X.W.); wuhaoxiang@gdaas.cn (H.W.); haochen@zju.edu.cn (H.C.); zhanyy2001@163.com (Y.Z.); 2 Key Laboratory of Genetics and Breeding of High Quality Rice in Southern China (Co-Construction by Ministry and Province), Ministry of Agriculture and Rural Affairs, Guangzhou 510640, China; 3 Guangdong Key Laboratory of New Technology in Rice Breeding, Guangzhou 510640, China; 4 Guangdong Rice Engineering Laboratory, Guangzhou 510640, China

**Keywords:** rice (*Oryza sativa* L.), appearance quality, milled rice grain shape, MGL, MGW, MLWR, QTL, RIL

## Abstract

Grain shape in rice (*Oryza sativa* L.) is a complex trait governed by multiple quantitative trait loci (QTLs). To dissect the genetic basis of rice shape, QTL analysis was conducted for milled rice grain width (MGW), milled rice grain length (MGL), and milled rice length-to-width ratio (MLWR) using a recombinant inbred line (RIL) population of F_10_ and F_11_ generations derived from a cross between Yuexiangzhan and Shengbasimiao. A high-density genetic map consisting of 2412 bins was constructed by sequencing 184 RILs, spanning a total length of 2376.46 cM. A total of 19 QTLs related to MGL, MGW, and MLWR were detected under two environments. The range of phenotypic variation attributed to individual QTL ranged from 1.67% to 32.08%. Among those, a novel locus for MGL, MGW and MLWR, designated as *qMLWR3.2*, was pinpointed within a specific ~0.96-Mb region. Two novel loci for MGW and MLWR, *qMLWR11.1* and *qMLWR11.2*, were verified within ~1.22-Mb and ~0.52-Mb regions using three RIL-developed populations, respectively. These findings lay the foundation for further map-based cloning and molecular design breeding in rice.

## 1. Introduction

Rice (*Oryza sativa* L.), as one of the most vital food crops, serves as a significant energy source for roughly half of the population in the world. As lifestyles improve, consumer expectations for rice quality have risen. Rice quality broadly encompasses processing, appearance, cooking, and eating quality and nutritional quality [1]. Among these, grain shape plays a pivotal role in determining appearance quality, yield, and market value. Grain shape can be further categorized by grain length (GL), grain width (GW), grain thickness (GT), and length-to-width ratio (LWR) [2]. High-quality rice with an appealing appearance significantly influences consumer willingness to pay and market value.

The grain shape of rice is a complex trait influenced by numerous genes and environmental factors. To date, some studies have counted more than quantitative trait loci (QTLs) related to rice grain shape have been identified [3]. According to statistics, more than 500 QTLs for grain size have been collected in the Gramene database (https://archive.gramene.org/qtl/, accessed on 6 October 2024), but only a few have been cloned. For grain length, *GS3* was the first cloned QTL in rice for grain shape, negatively regulating grain length by competitively interacting with RGB1 [4,5,6]. *GL3.1* encodes a protein phosphatase kelch (PPKL) family-Ser/Thr phosphatase and regulates cyclin T1;3 to control grain length [7]. *WTG1* primarily affects grain shape by influencing cell expansion, with overexpression leading to narrow, thin, long grains due to elongated cells [8]. *CLG1*, encoding an E3 ubiquitin ligase, inversely regulates grain length [9]. *GW2* [10,11], *gw2.1* (an allele of *GW2*) [12], *GW5*/*GSE5* [13,14], *GSW3.1* [15], *GS5* [16], *GW6*/*OsGASR7* [17,18], and *TGW2* [19] regulate grain width and weight in rice. *GSW3* encodes a GTPase-regulated protein and negatively regulates grain size and weight [20]. *GWY10* encodes a conserved actin protein, controlling grain width in model OsbZIP47–GWY10–CDKB2 [21]. The length-to-width ratio of grains is a critical trait regulated by grain length and grain width. Notably, *TGW3* (*OsSK41*/*OsGSK5*) is a negative regulator of grain length and weight [22,23]. GL7/GW7/SLG7 controls the length-to-width ratio, with upregulation increasing the seed length-to-width ratio in rice [24,25]. The dominant QTL *GSE9* influences grain length and width, contributing to shape differences between *indica*/Xian and *japonica*/Geng varieties [26]. In addition, transcriptional regulators play an important role in the regulation of rice grain shape, such as *GW8*/*OsSPL16* [27,28], *GLW7*/*OsSPL13* [29], *GS2*/*OsGRF4* [30,31], *GS9* [32], and *GL6* [33]. These findings underscore the complexity of the genetic and molecular mechanisms underlying grain shape in rice. Therefore, identifying the QTLs associated with grain shape is crucial for understanding the molecular basis of this complex trait in rice. Generally, genetic populations such as recombinant inbred lines (RILs), chromosome segment substitution lines (CSSLs), backcross inbred lines, introgression lines (ILs) and near-isogenic lines (NILs) have been developed to identify complex traits QTLs [3,34,35,36]. RILs are a collection of strains where parent strains are crossed to create recombinants that are then inbred to isogenicity, providing a permanent resource for trait mapping and analysis [37].

It is well known that the grain length or length-to-width ratio of *indica* rice is longer than that of *japonica* rice. Thus, as detailed above, some important shape QTLs and genes were identified and cloned from natural and genetic populations constructed from *indica* and *japonica* rice [34,35,38]. Nevertheless, the grain shape remains distinct in *indica*, which carries the major grain shape gene [39], indicating the potential existence of additional, as yet unidentified, minor grain shape genes in *indica*. Furthermore, recent studies have identified novel QTLs in *indica*/*indica* populations [40,41,42]. Consequently, the identification and cloning of novel QTLs for grain shape utilizing *indica*/*indica* genetic populations is a realistic proposition that constitutes a pivotal contribution to the comprehension of the molecular regulatory mechanisms underlying grain shape in rice. In order to discover the novel grain shape genes, QTL mapping of milled rice grain shape-related traits was conducted in two cropping seasons using a RIL population derived from *indica* varieties, Yuexiangzhan and Shengbasimiao, and a high-density genetic map. A total of 19 QTLs influencing milled rice grain width, length, and the length-to-width ratio were identified across both seasons. Moreover, the genetic effect of two novel quantitative trait loci (QTLs) (*qMLWR11.1* and *qMLWR11.2*) for milled grain width and milled grain length-to-width ratio were validated using three F_2_ populations. The findings presented herein provide a foundation for the cloning of *qMLWR11.1* and *qMLWR11.2* and may prove valuable in the breeding of new high-quality rice varieties.

## 2. Results

### 2.1. Phenotypic Variation of Parents and RILs

**Figure 1:**
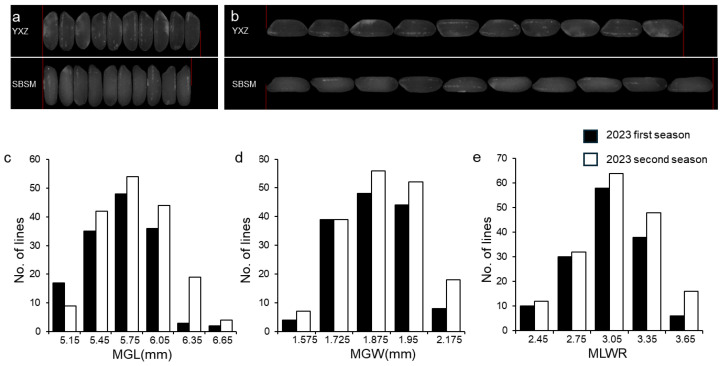
Phenotypic characteristics of milled rice shape of parents and RILs. (**a**,**b**) Grain phenotypes of two rice parents, Yuexiangzhan (YXZ) and Shengbasimiao (SBSM). Between the red lines indicate the total width and total length of the ten grains. (**c**–**e**) Frequency distribution of milled rice grain length (MGL), milled rice grain width (MGL), and milled rice length-to-width ratio (MLWR), in the 2023 first and second seasons.

To explore the genetic foundations of rice grain shape, two *indica* rice varieties, Yuexiangzhan and Shengbasimiao, were chosen as parents due to their marked differences in grain shape traits (Figure 1a,b, Table 1). These varieties were used to develop the RIL population. The milled rice grain width (MGW), milled rice grain length (MGL) and milled rice grain length-to-width ratio (MLWR) of the parents and 184 RILs were examined across two environments. Compared to Yuexiangzhan, Shengbasimiao exhibited larger values in MGL and MLWR and smaller values in MGW (Figure 1, Table 1). In two environments of repeated field trials, MGL ranged from 5.07 mm to 6.93 mm and from 5.12 mm to 6.70 mm, respectively. The ranges of MGW were 1.56–2.27 mm and 1.58–2.22 mm, and MLWRs were 2.31–3.67 and 2.38–3.82 mm (Table 1). Observations over two cropping seasons showed that all traits in the RIL population exhibited significant transgressive segregation and conformed to a normal distribution (Figure 1a–e), suggesting the involvement of multiple genes in these traits. Correlation analysis indicated a significant positive correlation between MLWR and MGL, and a significant negative correlation between MLWR and MGW (Figure S1).

### 2.2. Construction of Genetic Linkage Map

**Figure 2:**
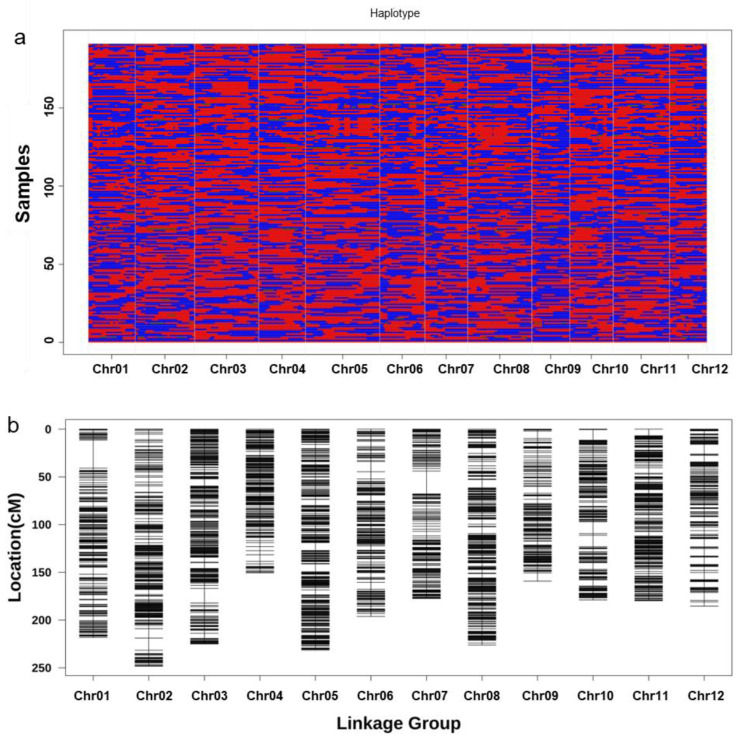
Bin map and genetic linkage map. (**a**) Bin map of 184 RILs. Red indicates the SBSM genotype, blue indicates the YXZ genotype, green indicates the heterozygous genotype, and gray indicates the deletion. The x axis shows the bins along the chromosomes. The y axis shows 184 lines. (**b**) Genetic linkage map.

For the RILs, 389,160 high-quality SNPs were identified utilizing a genotyping-by-sequencing (GBS) approach. A linkage map was constructed containing 2412 bins for the remaining RILs. The bins ranged in length from 30 kb to 3.0 Mb, with an average distance of 0.99 cM between them. Chromosome lengths ranged from 150.64 cM (chromosome 4 with 183 bins) to 248.23 cM (chromosome 2 with 231 bins) (Figure 2, Table S1).

### 2.3. Detection of QTLs for Grain Shape in RILs

**Figure 3:**
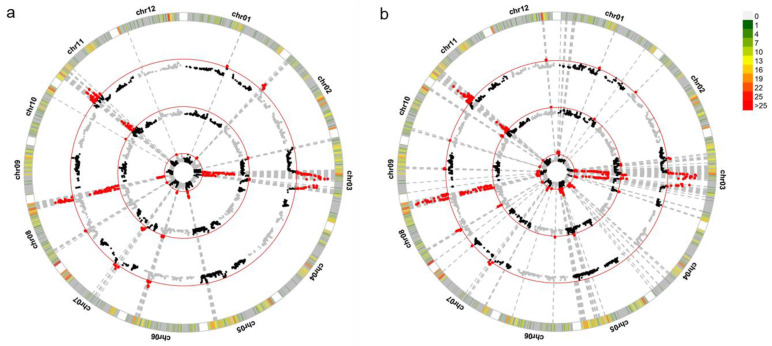
Genome-wide distribution of quantitative trait loci (QTLs) detected for three grain shapes in RILs of two environments. (**a**) The first season of 2023. (**b**) Second season of 2023. Rice chromosomes with bins are indicated in the outer circle. The outer to the inner circles represent MGL, MGW, and MLWR, respectively. For each bar diagram, the *x*-axis represents the physical location along each numbered chromosome. The *y*-axis represents the *P* value for the single-nucleotide polymorphism (SNP) association. Black and gray dots indicate the bins below the threshold, and red dots indicate the bins above the threshold. Red lines indicate the declaration thresholds.

The QTLs identified in the RIL population for milled rice grain shape traits are detailed in Table 2. A total of 19 QTLs related to MGL, MGW and MLWR were identified across two growing environments, distributed on chromosomes 2, 3, 4, 5, 6, 7, 8 and 11 (Figure 3). The contribution of each QTL to phenotypic variation ranged from 1.67% to 32.08% (Table 2).

For MGL, six QTLs were detected, and *qMGL3.1*, *qMGL3.2* and *qMGL8* were repeatedly detected in two growing environments. *qMGL3.1*, located between 16.20 Mb and 18.48 Mb on chromosome 3, demonstrated the most significant impact, explaining 15.80% of the phenotypic variance in the first season of 2023 and 25.28% in the second season. *qMGL3.2*, a newly identified QTL also on chromosome 3, explained 8.18% and 18.64% of the phenotypic variation in the 2023 first and second seasons, respectively. *qMGL8*, located between 26.98 Mb and 28.26 Mb on chromosome 8, contributed 5.24% and 4.08% of the phenotypic variation in the respective seasons. *qMGL3.1* and *qMGL3.2* showed negative additive effects, while *qMGL8* had positive additive effects. *qMGL4* and *qMGL5.1*, only detected in the 2023 second season, showed positive additive effects and contributed 6.54% and 6.22% to the phenotypic variation, respectively. *qMGL5.2*, only detected in the 2023 first season, contributed 6.68% of the phenotypic variation.

Six QTLs for MGW were identified, of which three were repeatedly detected in both growing environments. *qMGW8* emerged as the most impactful, explaining 27.02% and 32.08% of the phenotypic variation in the first and second seasons of 2023, respectively. *qMGW11.1* and *qMGW11.2* were located between 2.49 Mb–3.91 Mb and 4.11 Mb–5.42 Mb on chromosome 11, contributing 11.92% (14.33%) and 9.29% (11.13%) of the phenotypic variation in the 2023 first (second) season, respectively. *qMGW7*, only detected in the first season, had positive additive effects on MGW, contributing 2.58% of the phenotypic variation. *qMGW3.1* and *qMGW3.2*, only detected in the 2023 second season, also displayed positive additive effects, contributing 1.67% and 1.81% of the variation, respectively.

For MLWR, seven QTLs were detected, collectively accounting for a significant portion of the phenotypic variation: 56.99% in the first season and 73.61% in the second season. Among these, *qMLWR3.1*, *qMLWR3.2*, *qMLWR8*, *qMLWR11.1* and *qMLWR11.2* were repeatedly detected. *qMLWR3.1* was the QTL located between 16.10 Mb and 18.48 Mb on chromosome 3. It shared the same interval with the major QTLs *qMGL3.1* and *qMGW3.1*. *qMLWR3.1* contributed 10.21% and 15.38% of the phenotypic variation in the two seasons, respectively. *qMLWR3.2*, a newly identified QTL, contributed 7.59% and 11.38% of the phenotypic variation in the two seasons, respectively, and shared the same interval with the QTLs *qMGL3.2* and *qMGW3.2*. *qMLWR8*, the major QTL, contributed 12.52% and 19.61% of the phenotypic variation in the two seasons, respectively, and was co-located with *qMGL8* and *qMGW8* in the interval between 26.98 Mb and 27.19 Mb on chromosome 8. On chromosome 11, two new QTLs, *qMLWR11.1* and *qMLWR11.2*, contributed 11.69% and 7.43% of the phenotypic variation in the first season and 18.60% and 8.64% in the second season, respectively. Both *qMLWR11.1* and *qMLWR11.2* showed positive additive effects on MLWR. *qMLWR11.1* was co-located with *qMGW11.1* in the overlapping interval between 2.67 Mb and 3.89 Mb on chromosome 11. *qMLWR11.2* was co-located with *qMGW11.2* in the overlapping interval between 4.90 Mb and 5.42 Mb on chromosome 11. *qMLWR2* and *qMLWR6* were only detected in the 2023 first season, contributing 2.69% and 4.86% of the variation, respectively.

The 16.20–18.48 Mb interval on chromosome 3 had significant effects on MGL, MGW, and MLWR in two growing environments. The cloned gene *GS3* was located at the interval of this major QTL [43]. *qMGL8*, *qMGW8* and *qMLWR8* were co-located 26.98–27.19 on chromosome 8, and the cloned gene *GW8* was located at the interval of this major QTL [27]. Three novel QTLs for milled rice grain shape, *qMLWR3.2* (*qMGW3.2*, *qGL3.2*), *qMLWR11.1* (*qMGW11.1*) and *qMLWR11.2* (*qMGW11.2*), were identified in this study. *qMLWR11.1* and *qMLWR11.2* showed negative additive effects on MGW and positive additive effects on MLWR. *qMLWR11.1* contributes more to phenotypic variation. Therefore, *qMLWR11.1* was selected for further validation.

### 2.4. Validation of qMLWR11.1 and qMLWR11.2

**Figure 4:**
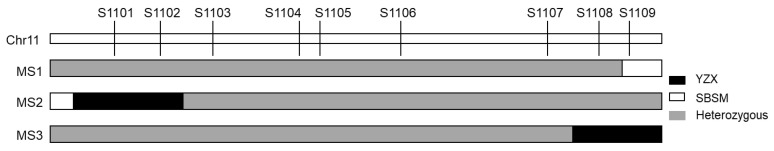
Genotype compositions of three populations.

Based on the results of QTL mapping, three derived populations (MS1, MS2, and MS3) came from the 184 RILs and were used to identify *qMLWR11.1* and *qMLWR11.2*. MS1 was heterozygous in the primary mapping region of *qMLWR11.1* (chromosome 11, S1101–S1106) and *qMLWR11.2* (chromosome 11, S1107–S1109) (Figure 4). MS2 was YXZ homozygous from marker S1101 to S1102 and heterozygous from marker S1103 to S1109. MS3 was heterozygous in the primary mapping region of S1101–S1107, and YXZ homozygous in the primary mapping region of S1108–S1109. MS1, MS2 and MS3 segregating populations consisting of 109, 123, and 116 individuals, respectively, were planted to validate the genetic effect of *qMLWR11.1* and *qMLWR11.2*. The effects of *qMLWR11.1* (S1101–S1106) in the three populations were similar, explaining 34.52–37.56% and 35.28–37.02% of the MGW and MLWR variations, respectively. On the other hand, the phenotypic variance explained by *qMLWR11.2* (S1107–S1109) was 21.86–30.11% for MGW and 26.29–27.92% for MLWR, respectively, being much lower than *qMLWR11.1* in three populations (Table 3). No QTL for MGL was detected in three derived populations, indicating that *qMLWR11.1* and *qMLWR11.2* influenced MLWR by controlling MGW.

## 3. Discussion

### 3.1. QTLs Dissect the Genetic Basis of Rice Grain Shape

Previous QTL localization studies typically constructed mapping populations derived from parents exhibiting significant phenotypic and genetic differences. The selected parents generally influence the efficiency of QTL detection, mapping, and cloning. Both YXZ and SBSM are slender-grain *indica* cultivars, with statistically significant variations in grain length, grain width and length-to-width ratio of milled rice. The milled rice grain shape variation of the RIL population was developed from a cross between SBSM and YXZ, which was extensive. The genetic basis of milled rice grain shape was discussed using this population, which is more conducive to identifying the new QTLs of milled rice grain shape. Six QTLs for MGL, six for MGW, and seven for MLWR were detected in two cropping seasons (Figure 3, Table 2). Some QTLs contain cloned genes, such as *qMGL3.1*, *qMGW3.1* and *qMLWR3.1* contain *GS3*, and new QTLs, such as *qMLWR11.1* and *qMLWR11.2*. Some QTLs controlling different traits were found within the same intervals, such as *qMGL3.1*, *qMGW3.1* and *qMLWR3.1*, co-located on 16.20–18.48 Mb of chromosome 3. *qMGL3.2*, *qMGW3.2* and *qMLWR3.2* were co-located on 21.28–22.24 Mb of chromosome 3. *qMGL8*, *qMGW8* and *qMLWR8* were co-located on chromosome 8. *qMGW11.1* and *qMLWR11.1* were co-located on 2.67–3.89 Mb of chromosome 11. *qMGW11.2* and *qMLWR11.2* were co-located on 4.90–5.42 Mb of chromosome 11. The co-localization of QTLs for MGL, MGW, and MLWR in the same genomic regions, with either the same or opposite directions of additive effects, likely contributed to the significant positive correlation between MGL and MLWR (correlation coefficients of 0.6* and 0.5*) and the significant negative correlation between MGW and MLWR (correlation coefficients of −0.8* and −0.8*). Moreover, the results are similar to previous studies [44], as the correlation coefficient between MGW and GLWR was larger than that between MGL and GLWR in these RILs, indicating that milled rice grain width has a greater influence on the length-to-width ratio. In this study, three, three and three QTLs of MGL, MGW and MLWR, respectively, were not repeated in the two cropping seasons. We performed a correlation analysis of the phenotypes in two cropping seasons, and the correlation coefficients of MGL, MGW and MLWR were 0.72, 0.84 and 0.88, respectively (Table S3). The interaction between phenotype and environment showed that environment had significant effects on MGW, MGL and MLWR. Therefore, there were some QTLs in our study that were not repeatedly detected in both environments.

### 3.2. Cloned Genes in the QTL Mapping Intervals

By analyzing chromosome positions, it was determined that *GS3*, which negatively regulates grain length, co-locates with *qMGL3.1*, *qMGW3.1* and *qMLWR3.1* [43]. GS3 inhibits the G protein signaling pathway by competitively interacting with RGB1, a G protein β subunit, thereby regulating grain length [5,6]. The interval for *qMGL5.2* includes the cloned gene *CLG*. *CLG1* encodes an E3 ligase that impacts grain size by targeting the Gγ protein GS3, a negative regulator of grain length [9]. The interval of *qMLWR6* contains a cloned gene, *TGW6*, which influences the timing of the transition from the syncytial to the cellular phase through the regulation of IAA supply, limiting cell number and grain length [45]. *qMGW8* and *qMLWR8* share the same interval, which includes the SBP-domain transcription factor *GW8/OsSPL16*, a positive regulator of cell proliferation [27]. *GW8/OsSPL16* is also involved in the repression of the *GW7* promoter, thereby regulating grain width [28]. In addition, *qHI-8*, the high harvest index QTL targeted by the RIL population in this study, was also identified at the *qMGW8* and *qMLWR8* locus [46]. These findings confirm the reliability of using RIL populations derived from YXZ and SBSM for rice QTL mapping. The identified QTLs containing cloned genes may be utilized in rice breeding to enhance appearance quality.

### 3.3. Grain Shape QTLs Were Newly Found and Verify

The formation of rice grain shapes is governed by complex genetic networks and environmental factors. In order to verify the genetic effect of QTL and conduct fine-mapping, near-isogenic lines (NILs) and heterogeneous inbred family (HIF) segregation populations are usually used [34,47]. In this study, three novel QTLs for milled rice grain shape, *qMLWR3.2* (*qMGL3.2*, *qMGW3.2*), *qMLWR11.1* (*qMGW11.1*), and *qMLWR11.2* (*qMGW11.2*), were detected repeatedly. *qMLWR3.2* effectively increased the grain width and reduced grain length and length-to-width ratio, while *qMLWR11.1* and *qMLWR11.2* were found to effectively reduce grain width and increase the length-to-width ratio of milled rice (Table 2). *qMLWR11.1* and *qMLWR11.2* were identified using three populations (MS1, MS2 and MS3) that came from 184 RILs with a heterozygous region in *qMLWR11.1* (S1101-S1106) and/or *qMLWR11.2* (S1107-S1109). These findings lay the groundwork for further detailed mapping of these QTLs and exploring their application in breeding programs aimed at improving the dimensions of white rice.

## 4. Materials and Methods

### 4.1. Plant Materials

**Figure 5:**
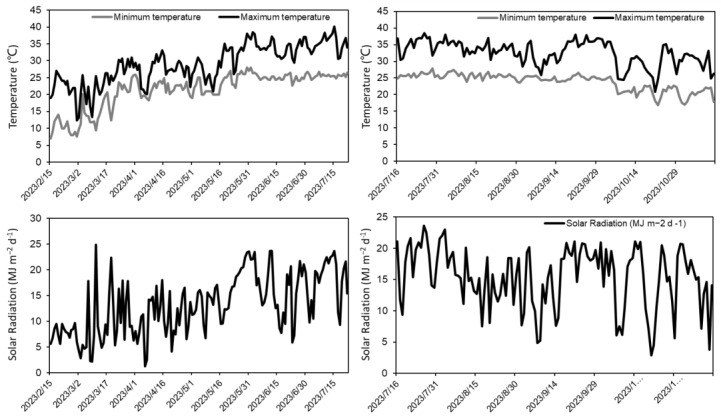
Maximum temperature, minimum temperature, and solar radiation in the first cropping season of 2023 in Baiyun, Guangdong Province, China.

Two *indica* cultivars, Yuexiangzhan (YXZ, No: Yueshendao1998001) and Shengbasimiao (SBSM, No: Yueshendao2005002), are widely planted in South China with similar growth periods. An RIL population comprising 184 lines was developed through the single-seed descent method from a cross between YXZ and SBSM. The RILs of F_10_ and F_11_ generations, along with their parent plants, were planted at the Baiyun Test Base of the Guangdong Academy of Agricultural Sciences (GDAAS), Guangzhou, Guangdong Province, China (113.44° E, 23.39° N, altitude 14.1 m) in two consecutive cropping seasons in 2023. The experiment field was sandy loam, and the nutrient composition of the soil was as follows: 0.73 g kg^−1^ total N, 0.74 g kg^−1^ total P and 63.6 g kg^−1^ total K. In the first cropping season of 2023, seeds were sown on 27 February after completing germination, and thirty-day-old seedlings were transplanted on 29 March, and harvested on 23 July. In the second cropping season of 2023, seeds were sown on 22 July, fifteen-day-old seedlings were transplanted on 6 August, and harvested on 12 November. Three independent lines were chosen from the RILs to validate the genetic effect of *qMLWR11.1* and *qMLWR11.2*, and these lines were developed for the independent F_2_ population, namely MS1, MS2 and MS3. Three F_2_ populations were planted at the Baiyun Test Base in the first cropping season of 2024, sown on February 26, and thirty-day-old seedlings were transplanted on March 27. All materials were planted in three rows, with six plants in each row at a spacing of 16.5 cm between plants and 26.5 cm between rows. We adhered to the local field cultivation and management practices. Partial meteorological data of the two cropping seasons in 2023 were statistically analyzed (Figure 5). The mean values of minimum temperature, maximum temperature and daily solar radiation were 21.3 °C, 29.1 °C and 13.2 MJ m^−2^ in the first cropping season and 23.9 °C, 32.5 °C and 15.4 MJ m^−2^ in the second cropping season, respectively.

### 4.2. Phenotyping and Statistical Analysis

Ten individuals from each line, exhibiting similar field performance, were selected and combined for harvest. The harvested grains were air-dried at room temperature (25 °C) for a minimum of three months to ensure a consistent moisture balance before measurement. The measurements for MGL, MGW, and MLWR were conducted after milling utilizing a scanner (ScanMaker *i*800*^plus^*, *MICROTEK*, Shanghai, China) and analyzed with *SmartGrain* software [48].

### 4.3. Genotyping and Genetic Map Construction

Genotyping of the parents and the 184 RILs was performed using the GBS approach on the Illumina HiSeq 2500 platform. For the parents Yuexiangzhan and Shengbasimiao, 8.3 Gb and 9.4 Gb of clean data were obtained, respectively, each achieving approximately 5× genome coverage. The 184 RILs collectively generated 53.8 Gb of clean data, also averaging 5× genome coverage. The sequencing data were aligned to the Nipponbare reference genome (https://rice.uga.edu/index.shtml, accessed on 23 December 2023). Following SNP calling and filtering, 389,160 high-quality, evenly distributed SNPs were selected for constructing the genetic map. These SNPs were utilized to identify bin blocks using the sliding window method. The resulting bin-based genetic linkage map was then generated using R/qtl software (https://rqtl.org/) [49].

### 4.4. QTL Analysis and Statistical Analysis

QTL analysis was conducted utilizing the composite interval mapping (CIM) method with WinQTLCart 2.5 software [50]. A logarithm of odds (LOD) threshold of 4 was set to determine significant QTLs, with intervals calculated to maintain a 95% confidence level. The grain shapes of two different homozygous genotypes within the same QTL were compared utilizing a *t*-test. A *p*-value of less than 0.05 indicated that the candidate gene was likely located within the heterozygous fragment. Otherwise, it was deemed to be within the homozygous fragment.

## 5. Conclusions

Grain shape is a critical quality trait in rice, influencing market preference and consumer acceptance. In this study, an RIL population was developed from a cross between the *indica* rice varieties Yuexiangzhan and Shengbasimiao. Utilizing this population, a high-density genetic map was constructed, featuring 2412 bin markers and spanning 2376.46 cM across 12 chromosomes. The average interval between markers was 0.99 cM. Over two cropping seasons, 19 QTLs influencing milled rice grain shape were identified. The range of phenotypic variation attributed to individual QTL ranged from 1.67% to 32.08%. Among these, a major QTL was discovered that simultaneously influenced MGL, MGW and MLWR, likely corresponding to the cloned gene *GS3*. Additionally, three novel QTLs affecting milled rice grain length, width and length-to-width ratio were identified, *qMLWR3.2*, *qLWR11.1* and *qLWR11.2*. *qMLWR3.2* was identified and narrowed to a ~1.5-Mb interval, influencing MGL, MGW, and MLWR. *qLWR11.1* and *qLWR11.2* were defined and verified in the interval of ~1.22 Mb and ~0.52 Mb, respectively, and are confirmed to control milled rice grain width and length-to-width ratio, showing positive additive effects on MLWR. These findings provide a foundational basis for advancing map-based cloning and molecular design breeding in rice.

## Figures and Tables

**Table 1:** Phenotypic analysis of milled rice grain-shape-related traits of parents and populations.

Trait	Parents		2023 First Season				2023 Second Season			
	YXZ	SBSM	Mean	SD	Max	Min	Mean	SD	Max	Min
MGL (mm)	5.3 ± 0.19	5.73 ± 0.18 **	5.72	0.33	6.93	5.07	5.83	0.34	6.70	5.12
MGW (mm)	2.16 ± 0.06	1.94 ± 0.06 **	1.89	0.14	2.27	1.56	1.91	0.15	2.22	1.58
MLWR	2.57 ± 0.11	2.85 ± 0.09 **	3.05	0.27	3.67	2.31	3.09	0.30	3.82	2.38

Note: YXZ, Yuexiangzhan; SBSM, Shengbasimiao; MGL, milled rice grain length; MGW, milled rice grain width; MLWR, milled rice grain length-to-width ratio. ** indicates the significant difference detected at *p* < 0.01 level.

**Table 2:** QTLs for milled rice shape traits in the RIL population derived from the cross between YXZ and SBSM.

Trait	Chr	Interval (Mb)	QTL	2023 First Season			2023 Second Season			*Gene*
				*p* Value	Effect	PVE (%)	*p* Value	Effect	PVE (%)	
MGL	3	16.20–18.48	*qMGL3.1*	3.78 × 10^−13^	−0.03	15.80	3.78 × 10^−13^	−0.04	25.28	*GS3*
MGL	3	21.28–24.44	*qMGL3.2*	2.37 × 10^−7^	−0.02	8.18	2.37 × 10^−7^	−0.03	18.64	
MGL	4	22.03–23.76	*qMGL4*				1.48 × 10^−5^	0.01	6.54	
MGL	5	21.30–23.29	*qMGL5.1*				6.31 × 10^−6^	0.01	6.22	
MGL	5	25.56–27.24	*qMGL5.2*	3.78 × 10^−5^	0.02	6.68				*CLG1*
MGL	8	26.98–28.26	*qMGL8*	1.19 × 10^−5^	0.01	5.24	1.82 × 10^−5^	0.03	4.08	*GW8*
MGW	3	16.20–18.49	*qMGW3.1*				4.09 × 10^−5^	0.01	1.67	*GS3*
MGW	3	21.28–22.24	*qMGW3.2*				1.48 × 10^−5^	0.01	1.81	
MGW	7	11.15–14.03	*qMGW7*	8.99 × 10^−5^	0.01	2.58				
MGW	8	25.35–27.19	*qMGW8*	8.82 × 10^−13^	0.02	27.02	4.44 × 10^−16^	0.03	32.08	*GW8*
MGW	11	2.49–3.91	*qMGW11.1*	1.08 × 10^−7^	−0.02	11.92	1.71 × 10^−8^	−0.03	14.33	
MGW	11	4.11–5.42	*qMGW11.2*	9.78 × 10^−6^	−0.01	9.29	1.55 × 10^−4^	−0.01	11.13	
MLWR	2	0–2.8	*qMLWR2*	3.28 × 10^−5^	0.03	2.69				
MLWR	3	16.10–18.48	*qMLWR3.1*	2.64 × 10^−12^	−0.03	10.21	3.91 × 10^−14^	−0.05	15.38	*GS3*
MLWR	3	21.28–22.75	*qMLWR3.2*	2.80 × 10^−9^	−0.02	7.59	3.01 × 10^−9^	−0.04	11.38	
MLWR	6	25.83–27.35	*qMLWR6*	3.89 × 10^−5^	−0.02	4.86				*TGW6*
MLWR	8	25.36–27.34	*qMLWR8*	1.17 × 10^−7^	−0.02	12.52	4.44 × 10^−16^	−0.06	19.61	*GW8*
MLWR	11	2.67–3.89	*qMLWR11.1*	8.16 × 10^−7^	0.02	11.69	4.98 × 10^−12^	0.06	18.60	
MLWR	11	4.90–5.48	*qMLWR11.2*	7.42 × 10^−7^	0.02	7.43	7.18 × 10^−6^	0.03	8.64	

MGL, milled rice grain length; MGW, milled rice grain width; MLWR, milled rice grain length-to-width ratio; Chr, Chromosome; QTL, quantitative trait loci; PVE (%), phenotypic variance explained by a given QTL; Effect, additive effect (the positive value means that YXZ allele increases the trait value).

**Table 3:** QTL effects of *qMLWR11.1* and *qMLWR11.2* detected in the three populations.

Population	Trait	Interval	LOD	Effect	PVE (%)
MS1	MGW	S1101–S1106	15.23	−0.02	37.56
		S1107–S1109	12.16	−0.04	30.11
	MLWR	S1101–S1106	18.28	0.03	36.07
		S1107–S1109	11.44	0.02	26.29
MS2	MGW	S1101–S1106	15.56	−0.04	34.52
		S1107–S1109	12.11	−0.03	21.86
	MLWR	S1101–S1106	16.16	0.02	37.02
		S1107–S1109	10.82	0.02	27.92
MS3	MGW	S1101–S1106	13.70	−0.03	36.68
		S1107–S1109	12.64	−0.03	28.24
	MLWR	S1101–S1106	15.72	0.02	35.28
		S1107–S1109	12.88	0.03	26.54

Note: MGW, milled rice grain width; MLWR, milled rice grain length-to-width ratio; LOD, logarithm of odds; Effect, additive effect; PVE (%), phenotypic variance explained by the QTL.

## Data Availability

The original contributions presented in the study are included in the Supplementary Material, further inquiries can be directed to the corresponding author.

## Supplementary Materials

The following supporting information can be downloaded at: https://www.mdpi.com/article/10.3390/plants13223178/s1, Figure S1: Correlation coefficients among three traits in the RIL population of two environments; Table S1: Number of SNPs and bins on different chromosomes; Table S2: Primers used in verification of *qMLWR11.1* and *qMLWR11.2*. Table S3 Analysis of interaction between environment and phenotype.
