# Supplementary material for: Genetic Dissection of Milled Rice Grain Shape by Using a Recombinant Inbred Line Population and Validation of qMLWR11.1 and qMLWR11.2
Source: Plants (Basel). 2024 Nov 13;13(22):3178. doi: 10.3390/plants13223178 (PMC11597858; doi:10.3390/plants13223178)
Supplement: Supplementary file 1 [file plants-13-03178-s001.zip › Supplementary/Figure S1.pptx]

## Slide 1
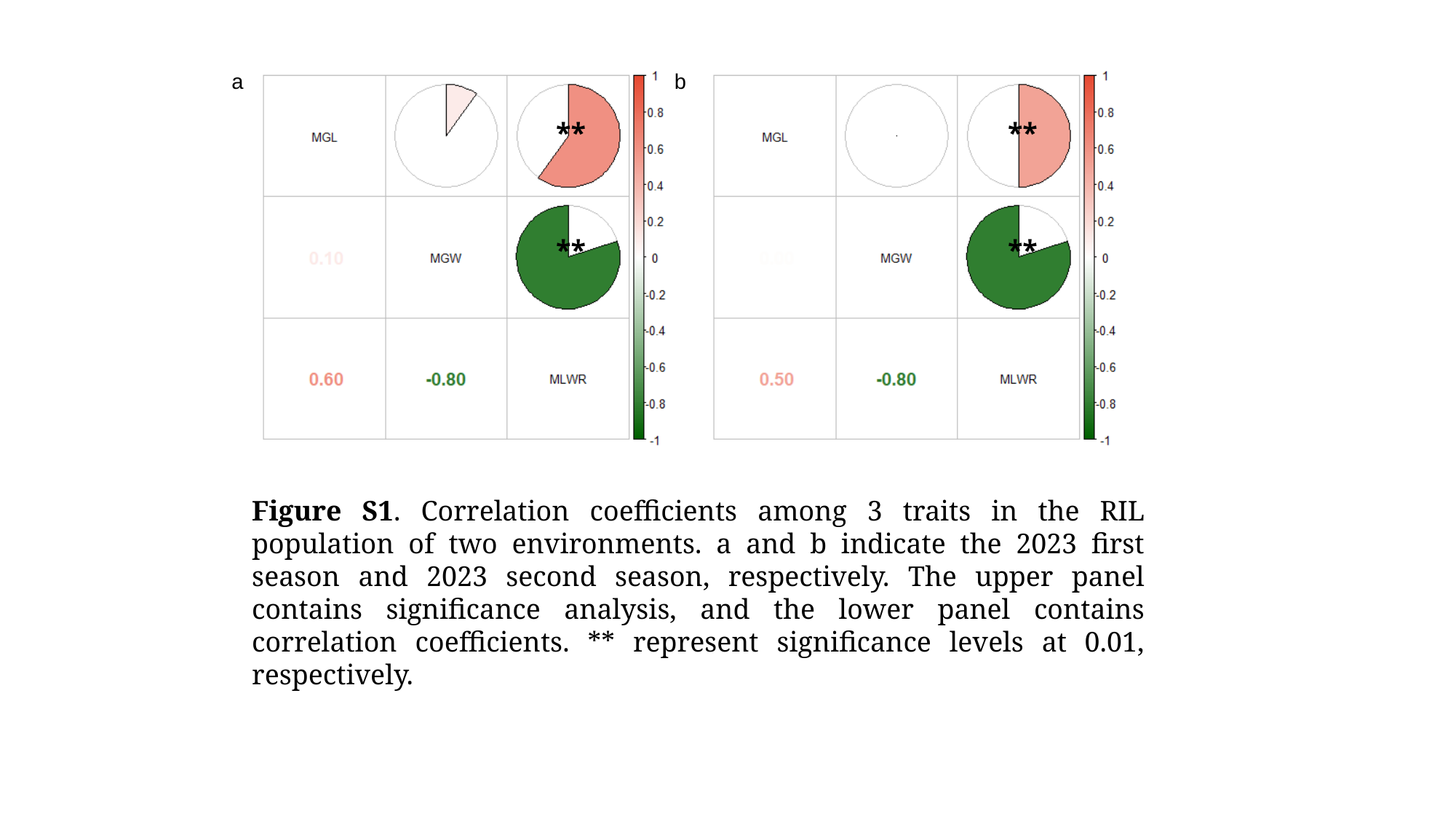

a
b
**
**
**
**
Figure S1. Correlation coefficients among 3 traits in the RIL population of two environments. a and b indicate the 2023 first season and 2023 second season, respectively. The upper panel contains significance analysis, and the lower panel contains correlation coefficients. ** represent significance levels at 0.01, respectively.
